# Sunk costs, psychological symptomology, and help seeking

**DOI:** 10.1186/s40064-016-3402-z

**Published:** 2016-10-03

**Authors:** David P. Jarmolowicz, Warren K. Bickel, Michael J. Sofis, Laura E. Hatz, E. Terry Mueller

**Affiliations:** 1 University of Kansas, 1000 Sunnyside Ave. 4050 Dole Center, Lawrence, KS 66045 USA; 2 Addiction Recovery Research Center, Virginia Tech Carilion Research Institute, Roanoke, VA USA

**Keywords:** Sunk cost, Logical fallacy, Health seeking, Depression, Binge eating disorder

## Abstract

Individuals often allow prior investments of time, money or effort to influence their current behavior. A tendency to allow previous investments to impact further investment, referred to as the sunk-cost fallacy, may be related to adverse psychological health. Unfortunately, little is known about the relation between the sunk-cost fallacy and psychological symptoms or help seeking. The current study used a relatively novel approach (i.e., Amazon.com’s Mechanical Turk crowdsourcing [AMT] service) to examine various aspects of psychological health in internet users (n = 1053) that did and did not commit the sunk-cost fallacy. In this observational study, individuals logged on to AMT, selected the “decision making survey” amongst the array of currently available tasks, and completed the approximately 200-question survey (which included a two-trial sunk cost task, the brief symptom inventory 18, the Binge Eating Scale, portions of the SF-8 health survey, and other questions about treatment utilization). Individuals that committed the fallacy reported a greater number of symptoms related to Binge Eating Disorder and Depression, being bothered more by emotional problems, yet waited longer to seek assistance when feeling ill. The current findings are discussed in relation to promoting help-seeking behavior amongst individuals that commit this logical fallacy.

## Background

Each day we make many decisions that impact our mental and physical health. For instance, we may choose whether or not to exercise, smoke cigarettes, or have that decadent desert. Each of these decisions is undoubtedly influenced by numerous variables, both immediate and temporally distant. Understanding these influences may help us facilitate healthy decisions across a wide range of populations.

The sunk-cost fallacy (effect) is one way that temporally distant events may influence current behavior (e.g., medical compliance; Christensen-Szalanski and Northcraft 1985). “The sunk cost effect is a maladaptive economic behavior that is manifested in a greater tendency to continue an endeavor once an investment in money, effort, or time has been made” (Arkes and Ayton 1999). The sunk-cost fallacy has been linked to psychological processes such as risk aversion (Northcraft and Neale 1986) foraging (Pavic and Passino 2011), and a desire to not appear wasteful (Arkes and Blumer 1985). Research on the sunk-cost fallacy, however, provides little guidance regarding which populations are most likely to commit the sunk-cost fallacy, and the relation between committing the sunk-cost fallacy and psychological health.

A few studies, however, have focused on identifying populations that do and do not commit the sunk-cost fallacy. For example, Strough et al. (2008) had younger adults (18–27 years old; n = 75) and older adults (58–71 years old; n = 73) read a series of vignettes about watching a boring movie. One pair of vignettes manipulated the cost of watching the movie (i.e., $10.95 or free), and the other pair of vignettes manipulated the time invested in watching the movie. Because the money/time spent on the movie cannot be recouped, watching the movie for longer after having paid for it, relative to when it is free, is an example of committing the sunk-cost fallacy. When asked how much longer they would continue to watch the movie, younger adults were more likely to commit the sunk-cost fallacy than older adults, particularly when the previous investment entailed money (also see Bruine de Bruin et al. 2007; Strough et al. 2011).

An individuals’ propensity or commit the sunk-cost fallacy may be elevated in certain clinical populations. First, rumination, a characteristic of depression (Nolen-Hoeksema 1991), entails an undue influence of past events over present behavior. Further, meditation, which is effective supplemental treatment for depression, has been shown to reduce the sunk cost fallacy (Hafenbrack et al. 2014). We therefore hypothesize that depressive symptoms may be positively related to ones’ propensity to commit the sunk cost fallacy. Specifically, because the sunk-cost fallacy involves previous investments (i.e., past events) irrationally influencing further investment (i.e., present behavior), individuals that commit the sunk-cost fallacy may also display more symptoms of depression. Second, the sunk-cost fallacy may provide helpful clues as to the underlying mechanism of overeating. In a study by Siniver, Mealem, and Yaniv (2013), for example, individuals who were forced to pay prior to getting access to an-all you can buffet ate significantly more than those who were forced to pay after the meal. Further, individuals with a higher BMI may also be more likely to commit the sunk-cost fallacy in a trial-based analog task (Sofis et al. 2015). By extension, we hypothesize that symptoms associated Binge Eating Disorder, which is often co-morbid with depression symptomology (e.g., Schulz and Laessle 2010), may also be associated with a higher propensity to commit the sunk-cost fallacy. Specifically, we hypothesize that in binge eating disorder, the tendency to continue eating (i.e., to further consume/invest) may be in part a function of how much has recently been consumed (i.e., initial investment). The current investigation examines the potential relation between symptoms of binge eating disorder and the sunk-cost fallacy.

Although few studies have linked the sunk-cost fallacy to help seeking, one study did link committing the sunk-cost fallacy to treatment decisions. Specifically, Coleman (2010) presented scenarios wherein subjects indicated if they would spend time attending a free treatment for their back pain (i.e., massage) or would go to sessions of an inferior treatment (i.e., chiropractor) in which they had already invested money, time, or effort. The amount of the previous investment varied across groups. Specifically, participants either invested less than market value, market value, or more than market value. Larger investments of money or effort were associated with an increased tendency to use the inferior treatment, whereas investments of time had little impact.

The current study sought to fill these gaps in our understanding of the relation between the sunk-cost fallacy and both psychological symptomology and help seeking. Specifically, in addition to replicating previous relations (i.e., age and the sunk cost fallacy, treatment utilization and the sunk-cost fallacy), the present study investigated the relation between committing the sunk-cost fallacy and symptoms of BED, depression symptomology, and various self-reported help seeking behaviors. A novel but readily available technology (i.e., Amazon.com’s Mechanical Turk [AMT] crowdsourcing service) was used to examine these relations in a large sample of Internet users.

## Methods

### Participants

The current study was presented to participants as a Human Intelligence Task (HIT) using AMT. AMT users could access the survey only if they were from the United States and if requesters had previously accepted at least 90 percent of their HITs. 1190 individuals meeting these criteria completed the survey. To ensure careful completion of the survey, HITs were not accepted if AMT users indicated that they failed to understand the task instructions, completed fewer than 80 % of the survey items, completed the survey in less than 800 s (where the mean completion time was 1472 s), or had non-varying patterns of responding on the survey’s delay discounting task (a pattern indicative of inattentive responding; delay discounting data not reported). Analyses presented from the current study are from 1053 participating AMT users with data that passed these screening criteria. Of those participants, 54 % were female, the average age was 31.76 years old (SD = 11.53), the group was generally well educated (2 % had less than a high school diploma, 10 % had a high school diploma, 35 % had attended some college, 8 % had Associates degrees, 31 % had Bachelors degrees, and 14 % had advanced degrees) and the median income was $23,750 (IQR = $7000, $48,750). See Table 1 for demographic information broken down by group.

**Table 1:** Data from individuals that did and did not commit the sunk-cost fallacy

	No sunk cost (n = 219)	Sunk cost (n = 834)	p	d
Brief symptom inventory				
Somatization	4.85 (4.93)	5.91 (5.06)	0.202	
Depression	*2.66* (*2.64*)	*2.94* (*2.94*)	*0.006*	*0.209*
Anxiety—general	2.82 (2.24)	3.10 (2.32)	0.115	
Anxiety—panic	3.95 (3.68)	4.46 (3.88)	0.077	
Binge Eating Scale	*8.82* (*2.29*)	*10.14 (2.37*)	*0.026*	*0.169*
SF-8 Health Survey				
Overall health	1.60 (1.13)	1.66 (1.03)	0.424	
Physical problems limit activity	0.73 (0.94)	0.71 (0.93)	0.798	
Physical health limit work	0.67 (0.97)	0.63 (0.87)	0.619	
Bodily pain	1.41 (1.10)	1.42 (1.10)	0.864	
Energy	1.53 (0.92)	1.63 (0.83)	0.091	
Physical/emotional limit social	0.89 (1.04)	0.94 (1.01)	0.510	
Bothered by emotional problems	*1.25* (*1.08*)	*1.42* (*1.13*)	*0.015*	*0.185*
Emotional problems limit work	0.81 (0.99)	0.89 (1.02)	0.302	
When do you seek medical help	*2.86* (*1.32*)	*3.06* (*1.24*)	*0.027*	*0.168*
Demographics				
Age	*34.84* (*13.10*)	*30.95* (*10.92*)	<*.001*	*0.297*
Median income (IQR)	*$26,250* (*$7000*–*48,750*)	*$21,250* (*$11,000*–*55,000*)	*0.001*	*0.251*
Percent female	55.71	54.45	0.739	
Years of education	14.66 (2.08)	14.51 (2.11)	0.333	
Body mass index	26.10 (6.24)	26.56 (7.75)	0.416	

Values in italics show statistically significant effects

### Materials

The data represent a subset of answers to questions in an approximately 200-question survey about health, social behaviors and decision-making. This survey included questions indicative of committing the sunk-cost fallacy, demographic information (e.g., age, gender, income, education, and smoking status), the Brief Symptom Inventory-18 (BSI-18), the Binge Eating Scale (BES), a measure on treatment utilization, and a measure of the participant’s perceived physical and mental health.

#### The sunk cost fallacy

Participants read a pair of vignettes almost identical to those used by Strough et al. (2008). Vignettes are the most common method used to evaluate the sunk cost effect (Arkes and Blumer 1985; Staw 1976). Typically, participants are told about a hypothetical scenario wherein they previously invested in a service or commodity which sets up a choice or decision scenario that is the dependent variable of interest. In the current study participants were asked, based on the context provided by the initial scenario, whether or not they will make an investment of time and how much of that investment they will make [for a review of the various forms of sunk cost, see Roth et al. (2014)]. In the current study, each participant experienced two initial hypothetical scenarios wherein the only difference is that in one of them you have paid for the activity and in the other scenario you did not. Specifically, the first of these two vignette options entailed paying to watch the movie (i.e., “You paid $10.95 to see a movie on pay TV. After 5 min, you are bored and the movie seems pretty bad”), whereas the second vignette entailed watching the movie for free (i.e., “You are watching a movie on TV. After 5 min, you are bored and the movie seems pretty bad”). After reading each vignette, the participants indicated how much longer they would watch the movie (i.e., stop watching entirely, watch for 10 more minutes, watch for 20 more minutes, watch for 30 more minutes, or watch until the end). A participant would demonstrate the sunk-cost fallacy by indicating that they would be willing to watch the movie longer after being told that they had already paid for it.

#### Brief Symptom Inventory-18 (BSI-18)

Participants answered 18 questions that assess their levels of somatization, depression, and anxiety symptoms (Derogatis 2001). These questions asked how much a particular symptom has bothered them over the previous 7 days. Symptoms indicative of each of the three domains were intermixed, and all responses were made on a 0 (not at all) to 4 (extremely) likert-type scale.

#### Binge Eating Scale (BES)

Participants indicated which statement out of a group of 3–4 statements best describes their eating patterns. For instance, one group included the following numbered statements: “I don’t feel any guilt or self-hate when I overeat”; “After I overeat, I occasionally feel guilt or self-hate”; “Almost all the time I experience strong guilt or self-hate when I overeat.” This process was repeated across 16 groups of statements, and the score across all 16 groups were weighted (see Gormally et al. 1982, for details) and summed to indicate participants’ BES score. Higher BES scores indicate more binge-eating symptomology.

#### Utilization of treatment

The survey asked about participants’ propensity to seek treatment after feeling ill. Specifically, the questionnaire asked “When you are ill you usually seek medical assistance,” and participants indicated “immediately”, “the next day”, 2–3 days after beginning to feel ill”, “4–7 days after beginning to feel ill”, “only after 1 week”, or “never”.

#### The SF-8 Health Survey (4-week recall)

The eight questions asked on the SF-8 Health Survey (Ware et al. 2001) were used to gauge the participants’ current health situation and overall quality of life (Lefante et al. 2005). Specifically, participants answered a series of questions using 5–6 point likert-type scales. These questions included: “Overall, how would you rate your health during the past 4 weeks?”; “During the past 4 weeks, how much did physical health problems limit your usual physical activities?”; “During the past 4 weeks, how much trouble did you have doing your daily work, both at home and away from home, because of your physical health?”; “how much bodily pain have you had during the past 4 weeks?”; “During the past 4 weeks how much energy did you have?”;”During the past 4 weeks, how much did your physical health or emotional problems limit your usual social activities with family or friends?”; “During the past 4 weeks, how much have you been bothered by emotional problems (such as feeling anxious, depressed, or irritable)?”; “During the past 4 weeks, how much did personal or emotional problems keep you from doing your usual work, school or other daily activities?”

### Procedures

To participate in the study, AMT users first accessed the survey by logging onto the AMT service and selecting the “Decision Making Study” HIT. Although the Virginia Tech Institutional Review Board deemed the current study exempt from review, participants read an overview of the study and indicated that they agreed to participate in the tasks by checking a box on the screen. Compensation was provided for timely and thoughtful completion of the survey. Participants received $2.50 for submission of the survey. Additionally, users could receive a bonus of $2.50 for careful and complete responses to the survey items.

### Data analysis

For the sunk-cost assessment, any pattern of responses wherein the participant was willing to watch the movie longer after having paid for it was indicative of committing the sunk-cost fallacy. The present data are presented as a binary measure if the participants did or did not commit the fallacy. In other words, a participant was considered to have committed the sunk-cost fallacy if they chose to invest more time watching TV only after they were told that had already paid money ($10.95) for the activity. This would be in comparison to the condition in which they were told their initial time spent watching TV was free. Results of the sunk-cost fallacy assessment were used to create groupings whereby our other measures were compared using two-tailed two-sample t tests.

Data from the BIS-18 were analyzed based on a four-factor model (Andreu et al. 2008). This model contained both the somatization and depression categories typically used (Derogatis 2001), but broke the anxiety categories into two factors (i.e., Anxiety-General, Anxiety-Panic). Dividing anxiety into these two factors maximized the potential to see relations between anxiety and the sunk-cost fallacy. Scores from each category were summed, and the mean score from those that committed the sunk-cost fallacy were compared.

Data from the BES were analyzed by weighting the scores from each of the 16 BES questions (Gormally et al. 1982), and totaling the weighted scores across items. Higher score indicated a higher probability of binge eating disorder. These total scores were then compared in individuals that did and did not commit the sunk-cost fallacy.

Each of the eight health questions was analyzed individually. Specifically, the means for each of the SF-8 health questions were compared in individuals whom did and did not commit the sunk-cost fallacy.

## Results

Data from several measures for individuals that did (n = 834) and did not (n = 219) commit the sunk-cost fallacy are summarized in Table 1. Consistent with previous studies (Bruine de Bruin et al. 2007; Strough et al. 2008, 2011), individuals that committed the sunk-cost fallacy tended to be younger than those that did not. Additionally, they reported being significantly more bothered by emotional problems, and reported waiting longer to seek medical attention when they were feeling ill, than individuals that did not commit the sunk-cost fallacy. Moreover, individuals that committed the sunk-cost fallacy had significantly lower incomes. Examining the data from the BSI-18, individuals that committed the sunk-cost fallacy displayed more signs of depression than those that did not. This relation, however, was not observed for somatization, or either of the two anxiety subscales. Similarly, individuals that committed the sunk-cost fallacy scored higher on the Binge Eating Scale than those that did not.

**Fig. 1:**
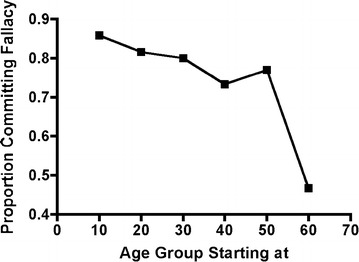
The proportion of individuals that committed the sunk cost at various age ranges (i.e., 10–19, 20–29, 30–39, 40–49, 50–59, 60+; labeled by the lowest age in the range)

Figure 1 provides a fine-grained analysis of the proportion of individuals in various age groups that committed the sunk-cost fallacy. Consistent with previous investigations, considerably fewer older adults (>60 years old) committed the sunk-cost fallacy than young adults (i.e., <20 years old). The decrease in proportion of individuals that committed the sunk-cost fallacy, however, was modest across most age brackets, with a robust decrease for older adults.

## Discussion

The present study linked the sunk-cost fallacy to reported urgency to seek medical treatment and to symptoms of depression. The current findings are an initial step towards delineating any relation between the sunk fallacy and health-oriented behavior (cf., Coleman 2010). Further, the current study replicates previous findings that older individuals commit the sunk cost less than younger individuals (cf., Bruine de Bruin et al. 2007; Strough et al. 2008, 2011), and provides initial evidence for associations between symptoms of depression and binge eating disorder. There are four additional points we would like to make about the current findings.

First, committing the sunk-cost fallacy was associated with the tendency to wait longer when seeking medical help due to illness in comparison those who do not commit the fallacy. Interestingly, individuals who committed the sunk-cost fallacy tended to wait longer to seek help when ill, and reported being more bothered by emotional problems, yet they reported that these emotional problems did not impact their social or occupational functioning (Ware et al. 2001). This may be because those who commit may be more likely to endure unpleasant conditions (i.e., further invest) is bolstered by their previous suffering (i.e., sunk costs). This possibility, however, awaits further research.

Nevertheless, with approximately 80 % of our sample committing the sunk-cost fallacy, the tendency of these individuals to suffer through illness may have wide-reaching implications for public health. Specifically, with such a large proportion of the population tending to suffer longer through physical and psychiatric distress, traditional methods of promoting the utilization of health care may be hindered. For example, traditional approaches to alleviating health disparities by providing free or low cost health insurance (i.e., minimal previous investment; Smith 2003) may not effectively compel this population (i.e., those that commit the sunk cost fallacy) to undergo the expense and effort (i.e., further investment) involved in utilizing health care. This possibility, which is supported by the observation that programs such as Medicaid have inefficiently promoted health (see Richman 2005, for a discussion), suggests that behavioral economic processes may need to be considered in the design of public health promotion programs.

The potential need to consider behavioral economic processes such as the sunk-cost fallacy when formulating approaches to health disparities may be compounded by the negative relation between committing the sunk-cost fallacy and socioeconomic status (i.e., income). Specifically, individuals that commit the sunk-cost fallacy have significantly lower incomes, and thus may be more subject to programs aimed at alleviating health disparities such as Medicaid. The precise relation between the tendency to commit the sunk-cost fallacy and utilization of programs aimed at decreasing health disparities, however, should be further investigated.

Second, individuals that committed the sunk-cost fallacy scored higher on assessments of symptoms of depression and binge eating than individuals who did not commit the fallacy. Consistent with the previous point, individuals with BED (Fairburn and Harrison 2003), and with depression (Wang et al. 2005; Young et al. 2001), are difficult populations to compel to utilize health services. The present findings, in combination with the age effect observed here and elsewhere (Strough et al. 2008, 2011), add to our understanding of which populations have a higher likelihood of committing the sunk-cost fallacy. From a practical standpoint, when dealing with populations with an elevated propensity to commit this logical fallacy, treatment retention strategies such as requiring a deposit (i.e., sunk costs) at the onset of treatment (Coleman 2010) may be particularly effective. These possibilities, however, await future research.

Third, the present study replicated previously observed relations, suggesting that AMT may be a reliable way to collect psychologically oriented data. For example, the negative relation between the tendency to commit the sunk cost fallacy and age was replicated in the present study (Strough et al. 2008, 2011). Moreover, Sprouse (2011) directly replicated one of his experiments on linguistic judgments using the AMT platform, and Bickel et al. (2004) recently replicated findings regarding the higher than average rates of delay discounting seen in cigarette smokers, relative to non-smoking controls, using AMT. Taken together, these findings suggest that the AMT platform produces data that are similar to those collected using more traditional laboratory-based methods.

Lastly, there are a number of issues that limit the conclusions of the present study. First, our sampling procedure yielded an unequal number of participants that did and did not commit the sunk-cost fallacy. Although this process clarified the proportion of a large sample that committed the sunk-cost fallacy, equal sample sizes in each group would have been ideal. Second, the order of the two sunk-cost questions was fixed (i.e., not counterbalanced). Although this could have impacted the present findings, previous investigations presenting these questions in counterbalanced order (Strough et al. 2008, 2011) did not report significant order effects. Third, the present study consisted of self-report data taken from a cross-sectional sample. Future research employing experimental analogs of the sunk cost fallacy and/or independent verification of psychological symptomology may strengthen the conclusions that can be made. Fourth, it is possible participants may have not attended to one or both of the vignettes used to evaluate the sunk-cost fallacy in the present study. The sunk cost effects observed, however, were systematic and in line with hypothesized relations with mental health symptoms (e.g., depression), symptoms of binge eating, and those with delayed treatment seeking, making such a confound less likely to have influenced the data. Finally, unlike previous studies, the current study only asked about sunk costs involving money rather than both money and time. Although asking about both types of investment may have improved our ability to observe effects, previous research has found that investments of money more reliably elicit the sunk cost fallacy than those of time (Coleman 2010).

## Conclusions

The present findings advance our understanding of the relation between the sunk-cost fallacy and health behavior. The current findings, however, await their application to the improvement of health behavior through their incorporation into individualized treatment (e.g., deposits for health services; Coleman 2010), and consideration when making decisions about decreasing health disparities (e.g., Medicaid; Richman 2005). These applications will test the utility of understanding the impact of sunk costs.
